# The complete methylome of an entomopathogenic bacterium reveals the existence of loci with unmethylated Adenines

**DOI:** 10.1038/s41598-018-30620-5

**Published:** 2018-08-14

**Authors:** Amaury Payelleville, Ludovic Legrand, Jean-Claude Ogier, Céline Roques, Alain Roulet, Olivier Bouchez, Annabelle Mouammine, Alain Givaudan, Julien Brillard

**Affiliations:** 1 DGIMI, INRA, Univ. Montpellier, Montpellier, France; 2 LIPM, Université de Toulouse, INRA, CNRS, Castanet-Tolosan, France; 3 GeT-PlaGe, INRA, US 1426, Genotoul, Castanet-Tolosan, France; 4 Present Address: Department of Fundamental Microbiology, Faculty of Biology and Medicine, University of Lausanne, Quartier UNIL/Sorge, Lausanne, CH1015 Switzerland

## Abstract

DNA methylation can serve to control diverse phenomena in eukaryotes and prokaryotes, including gene regulation leading to cell differentiation. In bacteria, DNA methylomes (i.e., methylation state of each base of the whole genome) have been described for several species, but methylome profile variation during the lifecycle has rarely been studied, and only in a few model organisms. Moreover, major phenotypic changes have been reported in several bacterial strains with a deregulated methyltransferase, but the corresponding methylome has rarely been described. Here we report the first methylome description of an entomopathogenic bacterium, *Photorhabdus luminescens*. Eight motifs displaying a high rate of methylation (>94%) were identified. The methylome was strikingly stable over course of growth, but also in a subpopulation responsible for a critical step in the bacterium’s lifecycle: successful survival and proliferation in insects. The rare unmethylated GATC motifs were preferentially located in putative promoter regions, and most of them were methylated after Dam methyltransferase overexpression, suggesting that DNA methylation is involved in gene regulation. Our findings bring key insight into bacterial methylomes and encourage further research to decipher the role of loci protected from DNA methylation in gene regulation.

## Introduction

DNA methyltransferases (MTases) are enzymes that catalyze the transfer of a methyl group from the universal methyl donor S-Adenosylmethionine (SAM) to a nucleotide^1^. MTases are widespread from eukaryotes to prokaryotes: 5-methylcytosine (m5C) and N^6^-methyladenine (m6A) methylation marks have been described in eukaryotes^2,3^ whereas additional N^4^-methylcytosine (m4C) marks can be detected in bacteria and archaea^4,5^. For years, DNA methylation studies mostly used whole genome bisulfite sequencing (WGBS), which only detects m5C^6^, until the advent of Single Molecule Real Time (SMRT) sequencing made it possible to also detect m4C and m6A modifications^7^.

DNA methylation in bacteria is involved in many cellular processes. It provides a defense against foreign DNA in restriction-modification systems (RM), where a restriction endonuclease (REase) acts in coordination with a DNA MTase. The MTase methylates self-DNA whereas exogenous DNA is cleaved by the cognate REase due to different methylation patterns. RM-MTases are classified into 4 different groups based on biochemical properties (e.g. their subunit organization, their recognition of palindromic vs asymmetric target DNA sequences…)^8^. The REBASE database is designed to identify REases and MTases and currently lists thousands of putative RM-MTases^9^.

Many studies, particularly in *Escherichia coli*, have described the role of DNA methylation in discriminating between the parental and newly-synthesized strand using the DNA mismatch repair (MMR) system^10^. During replication, errors may occur, and the MMR system excises the wrong base on the newly-synthesized unmethylated DNA strand^10^. The MMR system requires Dam (DNA adenine MTase), an MTase that is not coupled with a restriction enzyme and so called “orphan” or “solitary” MTase^5^. In *Gammaproteobacteria*, Dam methylates 5′-GATC-3′ motifs. Dam is also involved in epigenetic mechanisms by strongly contributing to the regulation of several genes expression^5^. In *E*. *coli* for example, the Lrp regulator can bind sites containing GATC sequences in the *pap* operon promoter region^11^. Lrp has greater affinity to unmethylated sites than fully-methylated (methylation on both strands) sites, and the competition between Dam and Lrp binding gives rise to two sub-populations, one expressing the Pap pilus and the other not^12^. Deregulation by mutation or overexpression of Dam in several bacterial species can lead to drastic phenotypic changes. For example, Dam overexpression leads to a decrease in virulence for *Yersinia pseudotuberculosis*, *Vibrio cholerae*, *Salmonella enterica*, *Pasteurella multocida*, *Aeromonas hydrophila* and *Photorhabdus luminescens*^13–18^. Dam mutants have also been described as showing impaired virulence in *S*. *enterica*, *Haemophilus influenzae*, *Klebsiella pneumoniae*, *A*. *actinomycetemcomitans* and *Y*. *pestis*^19–23^. Dam mutants are not viable in *Y*. *pseudotuberculosis* and *V*. *cholera*^16^ or suspected to be unviable in other bacterial species^18^. DNA cytosine MTase (Dcm) is another solitary MTase that is well conserved in *Gammaproteobacteria*^24^. The *dcm* gene is associated to *vsr* which encodes the very-short-patch repair system involved in T/G mismatches correction^24^. Dcm adds a methyl group to the second cytosine of CCWGG motifs. This solitary MTase has been shown to be involved in drug resistance, translation^25^ and stationary phase gene expression^26^. In addition to Dam, the best known example of solitary MTase involved in epigenetic mechanisms in bacteria^27^ is the CcrM (Cell cycle regulated MTase) found in many *Alphaproteobacteria*. Its main role in *Caulobacter crescensus* is regulation of the cell cycle and cell division^28,29^. It is essential for cell viability in fast-growing conditions^30^ but not in slow-growing conditions such as minimal media^31^. It has not been described as involved in MMR^31^. Many other solitary MTases are present in many bacterial genomes, but their role has not yet been described^32^.

SMRT sequencing can now detect all DNA methylation marks in genomes, opening opportunities to detect new methylated motifs^7^. This new-generation sequencing technology has been used to describe the methylome of several microorganisms. In bacteria, there has been a strong focus on animal pathogens, but the methylomes of some plant pathogens have also been reported^32–41^.

*P*. *luminescens* TT01 is an entomopathogenic bacterium member of the *Enterobacteriaceae*. It is found in symbiosis with a soil nematode from the genus *Heterorhabditis*. This nematobacterial complex is able to kill many crop-pest insects and can be used in biocontrol^42^. During its lifecycle, this bacterium has to switch between mutualism (within the nematode’s gut) and a pathogenic state (in the insect). This switch is controlled by a promoter inversion that results in Mad pilus expression^43^. However, phenotypic heterogeneity among a *P*. *luminescens* clonal population caused by an as-yet-unknown mechanism is also critical during another step of the bacterial lifecycle. It was recently demonstrated that only a minority (<1%) of the whole bacterial population is responsible for virulence in insects, as it is able to resist the antimicrobial peptides (AMPs) produced by the insect host^44^. The fact that this AMP-resistant subpopulation displays no genetic change compared to the wild-type population has raised the hypothesis that an epigenetic mechanism is involved in the occurrence of this subpopulation^44^. Moreover, we recently showed that overexpression of the *P*. *luminescens* Dam MTase decreases motility and virulence yet enhances biofilm formation^18^. However, the impact of this MTase overexpression on DNA methylation pattern remains to be investigated.

The aim of this study was to provide the first description of the full methylome of *P*. *luminescens* during different growth phases. We also investigated whether changes in DNA methylation pattern occur within the AMP-resistant subpopulation responsible for insect virulence, or after the Dam overexpression that leads to major phenotypic changes in *P*. *luminescens*. The various methylomes analyzed here led to the identification of eight methylation motifs, and open new fields of investigation into the role of DNA methylation in bacterial gene regulation.

## Results

### Predicted MTases in *P. luminescens* TT01

The *P*. *luminescens* TT01 genome harbors 47 genes which are annotated as methyltransferase or methylase, most of them encoding putative RNA MTases or protein MTases. Only 8 genes (plu0087, plu0338, plu0600, plu2710, plu2942, plu3417, plu3449 and plu3462) are annotated as DNA methyltransferase or DNA methylase^45^. Analysis of the *P*. *luminescens* TT01 genome using REBASE revealed 12 putative DNA MTase-encoding genes, i.e. the 8 genes cited above, plus plu0233, plu1935 and plu4197 which are annotated as encoding CHP (conserved hypothetical proteins) or unknown proteins, and plu4319 which is annotated as encoding a “Type I site-specific deoxyribonuclease HsdM”. Prediction of the protein domains revealed an S-adenosyl-L-methionine-dependent methyltransferase domain (Interpro domain IPR029063) in all of the 12 MTases.

**Figure 1:**
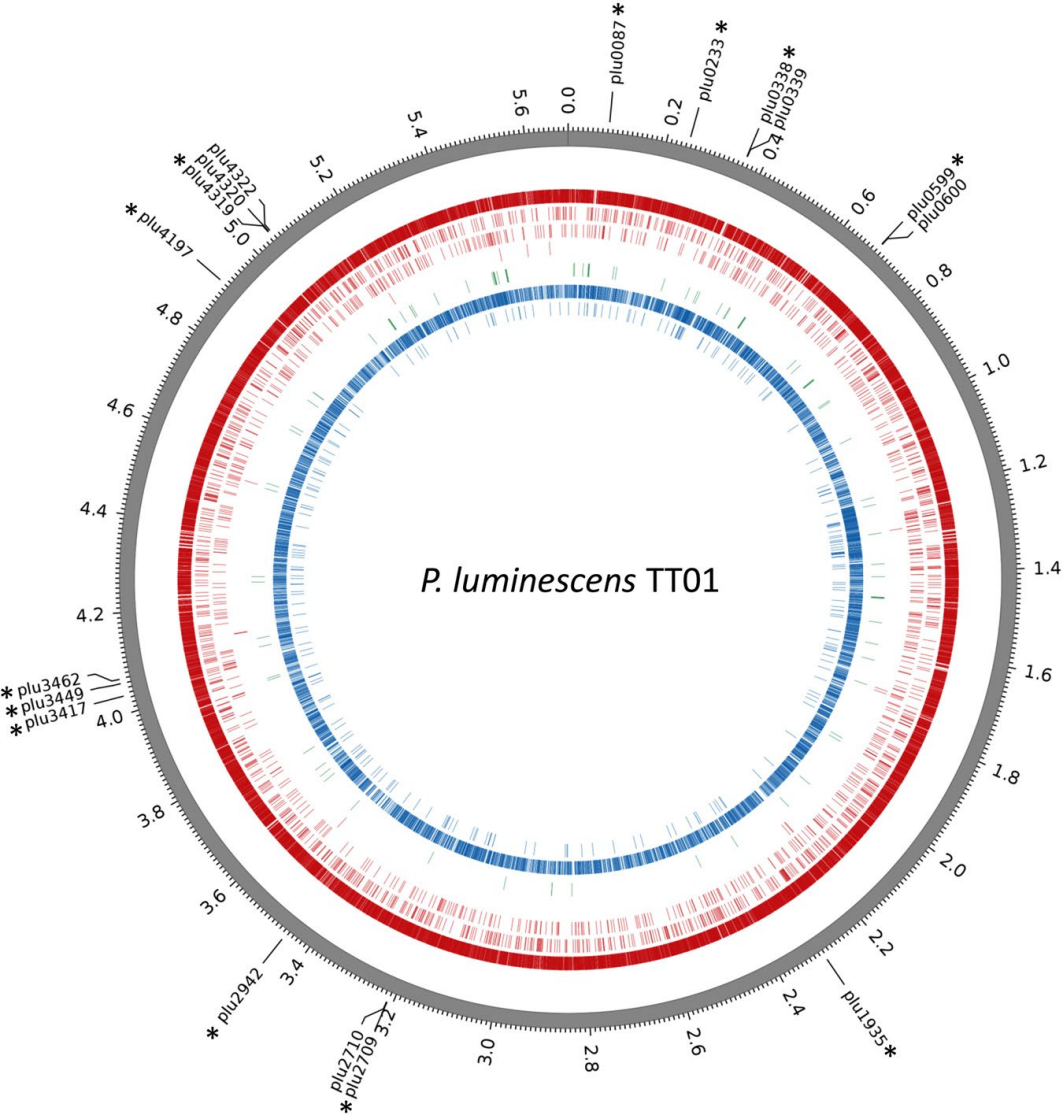
Circos plots displaying the distribution of methylated bases over the *P*. *luminescens* TT01 chromosome. Outermost track displays the genomic positions in megabases. The colored tracks display the location of the modification marks detected in at least one of the growth conditions tested, for each of the 8 identified motifs. Different colors represent different methylation types. From outer to inner: (red, m6A) GATC, GGANNNNNNRTGA/TCAYNNNNNNTCC, TGGCCA, CTCGAG; (green, m4C) AGGCCT; (blue, m5C) CCWGG, GGCGCC. Tick marks on the outermost track display the genomic positions of the 12 MTase-encoding genes (labelled with an asterisk) and their 5 cognate partners; see Table 1 for details. Note that the *P*. *luminescens* TT01 strain harbors no plasmid.

The 12 putative DNA MTase-encoding-genes found in *P*. *luminescens* TT01 are listed in Table 1. While 4 MTases are associated with REases, 7 are solitary MTases, and one is a hybrid MTase. The 12 loci were located all over the chromosome (Fig. 1). One predicted MTase (plu4319) corresponded to a Type I^9^ while the remaining MTases were presumed to classify as Type II. For 8 of them, REBASE analysis proposed a recognition sequence (Table 1)^9^.

**Table 1:** List of putative *P*. *luminescens* TT01 MTase encoding genes.

Gene name or Label	Protein label	Interpro Domains	MW (Da)	Type	Cognate partner^a^	Target Sequence^b^	Genome Position	Genomic Location^c^
*dam* (*plu0087*)	M.PluTDam	IPR012327, IPR012263, IPR029063, IPR023095, IPR002052	31567	Solitary		Gm6ATC (motif I)	81849–82661	Core Genome
*plu0233*	PluTORF233	IPR002052	121133	Hybrid		?	242879–246085	RGP5
*dcm* (*plu0338*)	M.PluTORF0338	IPR001525, IPR029063, IPR018117, IPR031303	54918	Solitary	*plu0339*,Vsr	Cm5CWGG (motif VI)	361215–362651	GI9
*plu0599*	M.PluTI	IPR001525, IPR029063	42313	II/R-M	*plu0600*, PluTI	GGm5CGCC (motif VII)	679941–682068	Core Genome
*plu1935*	M.PluTORF1935	IPR002941, IPR029063, IPR001091, IPR002052	78939	II/R-M	*plu1934*, PluTORF1935	AGGCm4CT (motif IV)	2301611–2303698	RGP46
*plu2709*	M.PluTORF2709	IPR029063, IPR025931, IPR025931	27200	II/R-M	*plu2710*, PluTORF2710	CTCGm6AG (motif V)	3211074–3213464	RGP66
*plu2942*	M.PluTORF2942	IPR007757, IPR029063	24665	Solitary		Gm6ATC	3452873–3453517	P71
*plu3417*	M.PluTORF3417	IPR002941, IPR029063, IPR001091	21450	Solitary		?	4034728–4035285	P80
*plu3449*	M.PluTORF3449	IPR012263, IPR029063, IPR023095	29802	Solitary		?	4059699–4060469	P80
*plu3462*	M.PluTORF3462	IPR002941, IPR001091, IPR029063, IPR002052	18578	Solitary		?	4068792–4069265	P80
*plu4197*	M.PluTORF4197	IPR009528, IPR029063	18824	Solitary		CTGCm6AG	4911020–4911514	GI94
*hsdM* (*plu4319*)	M.PluTORF4319	IPR003356, IPR022749, IPR004546, IPR029063, IPR002052	57671	I/R-M	*hsdS* (*plu4320*), S.PluTORF4319, *hsdR* (*plu4322*), PluTORF4319	GGm6AN6RTGA(motif IIIa)/TCm6AYN6TCC (motif IIIb)	5043966–5044616	GI95

^a^Gene name and protein label of cognate partners (if any) is indicated.

^b^Predicted target sequence is indicated. Motifs identified in this study are underlined (with their number in brackets).

^c^As described by Ogier *et al*.^46^. GI, genomic islands; RGP, regions of genomic plasticity; P, phagic regions.

### Distribution of predicted MTases in Gram-negative bacteria

The genomic context and taxonomic distribution of putative *P*. *luminescens* TT01 MTase-encoding genes are presented in Fig. S1. Most (n = 10) of the MTases encoding loci were located in the accessory genome and are associated with phagic regions (n = 4), genomic islands (n = 3), or regions of genomic plasticity (n = 3) (Table 1). The *dam* gene is located in the core genome and an ortholog was present in each of the *Photorhabdus* and *Xenorhabdus* (a closely related genus, with similar lifestyle^46^) complete available genomes, with a conserved synteny (Fig. S1). *dam* was also broadly distributed among many (>100) organisms of the *Enterobacteriaceae* family (Fig. S1), suggesting that this gene was ancestral in the genome. In contrast, plu0599, encoding M.PluTI^47^, is also located in the core genome (Table 1) but only conserved in two other strains of *Xenorhabdus* and *Photorhabdus* (Fig. S1). Other *P*. *luminescens* TT01 MTase-encoding genes were variably distributed among *Enterobacteriaceae*. For example, plu4197 was only found in *P*. *luminescens* TT01 genome whereas putative orthologs of several MTases (*dcm*, plu2942, plu3417, plu3449, plu3462, plu4319) were found in more than 50 organisms of the *Enterobacteriaceae* family (Fig. S1). The variable taxonomic distributions of MTases encoding genes and their high prevalence in the accessory genome, including an association with phage or DNA recombination genes, suggest that most of these genes have been acquired by horizontal gene transfer.

### Identification of Methylation Motifs in *P. luminescens* Genome

In order to have a complete overview of all the existing methylation sites over the *P*. *luminescens* genome, two complementary sequencing technologies were used: (i) SMRT sequencing by PacBio which can identify m6A and (to a lesser extent) m4C, and (ii) WGBS by Illumina (MiSeq) which can identify m5C.

The methylome analysis was first performed on DNA samples extracted from WT *P*. *luminescens* cells harvested during the mid-exponential-growth phase (OD = 0.3–0.4, hereafter referred to as EP for exponential phase). The SMRT sequencing yielded an average coverage of 182X allowing the identification of a high number (n = 41143) of statistically significant (QV score ≥30, see the Methods section for details) DNA modification marks and the identification of 6 conserved motifs: GATC; TGGCCA; GGANNNNNNRTGA; TCAYNNNNNNTCC; AGGCCT; CTCGAG (Table 2). Five of them display m6A modifications and one displays an m4C modification. The WGBS approach allowed the discovery of additional m5C modification marks (n = 10898), grouped in two motifs: CCWGG and GGCGCC (Table 3). Altogether, we found 52041 methylated nucleotides distributed all over the *P*. *luminescens* genome (Fig. 1). For convenience, the 8 identified motifs were numbered according to sequencing technology used and their occurrence in the *P*. *luminescens* TT01 genome, and are hereafter referred to as motifs I to V for SMRT, with partner motifs GGANNNNNNRTGA/TCAYNNNNNNTCC numbered IIIa and IIIb, respectively, and motifs VI and VII for WGBS (Tables 2 and 3). The number of methylated motifs found in the genome ranged from 28 (motif V) to 37465 (motif I). The proportion of a given motif found methylated in the *P*. *luminescens* TT01 genome ranged from 94.0% (motif VII) to 100% (motif V).

**Table 2:** Motifs detected by SMRT sequencing in *P*. *luminescens* TT01*.

Motif	Fraction	N Detected	Mean Score	Mean IPD Ratio	Mean Coverage
**Motif I** **GATC** (37500)					
EP	0.999	37465	213.9	5.28	148.3
LE	0.999	37445	146.2	5.34	93.4
SP	0.999	37466	109.0	6.18	63.8
LS	0.999	37476	177.0	5.19	120.0
AMP-R	0.999	37444	120.9	5.64	74.6
Control	0.998	37439	136.7	5.61	83.5
DAM+	>0.999	37496	211.2	5.23	141.0
**Motif II** **TGGCCA** (1256)					
EP	0.998	1254	205.0	5.37	149.7
LE	0.999	1255	136.0	5.30	94.6
SP	0.998	1254	100.9	6.09	63.9
LS	0.998	1253	161.6	5.04	120.0
AMP-R	0.998	1253	116.0	5.74	75.6
Control	0.998	1254	127.2	5.68	84.6
DAM+	1.000	1256	194.2	5.13	143.8
**Motif IIIa/IIIb** **GGANNNNNNRTGA** (1112)/**TCAYNNNNNNTCC** (1112)					
EP	0.997	1109	207.3	5.27	147.2
	0.996	1107	189.8	4.61	145.4
LE	0.994	1105	125.9	4.48	92.3
	0.996	1108	138.1	5.18	93.2
SP	0.994	1105	96.2	5.23	63.1
	0.998	1110	102.4	5.93	63.4
LS	0.988	1099	147.9	4.19	120.2
	0.996	1108	167.6	4.98	121.1
AMP-R	0.993	1104	108.7	4.91	73.7
	0.996	1108	116.0	5.57	74.3
Control	0.994	1105	119.2	4.78	81.5
	0.997	1109	128.8	5.48	83.4
DAM+	0.992	1103	174.0	4.28	139.2
	0.997	1109	194.3	5.01	140.6
**Motif IV** **AGGCCT** (184)					
EP	0.978	180	152.0	3.76	154.3
LE	0.978	180	98.6	3.78	93.8
SP	0.978	180	75.4	3.94	61.7
LS	0.973	179	117.4	3.82	110.7
AMP-R	0.962	177	89.6	3.82	78.7
Control	0.984	181	96.4	3.83	87.7
DAM+	0.989	182	143.1	3.69	152.9
**Motif V** **CTCGAG** (28)					
EP	1.000	28	200	5.44	151
LE	1.000	28	137	5.26	93.4
SP	1.000	28	99.7	6.65	59.8
LS	1.000	28	160.9	5.21	122.4
AMP-R	1.000	28	77.6	6.25	47
Control	1.000	28	134	5.89	86.1
DAM+	1.000	28	214	5.27	156

*Methylated base is underlined (m6A or m4C). The number of each motif found in the genome is indicated in brackets. EP, exponential phase; LE, late exponential phase; SP, stationary phase; LS, late stationary phase; AMP-R, polymyxinB-resistant subpopulation; Control, *P*. *luminescens* TT01 harboring the pBBR1MCS5 empty plasmid; Dam+, Dam overexpressing *P*. *luminescens* strain harboring the pBB-Dam plasmid. IPD, interpulse duration.

**Table 3:** Motifs detected by bisulfite sequencing in *P*. *luminescens* TT01*.

Motif	Fraction	N Detected	Mean Coverage
**Motif VI** **CCWGG** (10998)			
EP	0.948	10428	51.9
SP	0.953	10486	49.9
**Motif VII** **GGCGCC** (500)			
EP	0.940	470	56.8
SP	0.942	471	50.5

*Methylated base is underlined (m5C). The number of each motif found in the genome is indicated in brackets. EP, exponential phase; SP, stationary phase.

### Methylation motifs are conserved during various growth conditions

In order to determine whether the methylation pattern can evolve over the course of growth, methylome analysis after SMRT sequencing was also performed on bacterial cells harvested during other growth conditions: WT cells harvested during the late exponential-growth phase (OD = 0.9, hereafter referred as LE), stationary phase (overnight growth reaching OD = 1.5, hereafter referred as SP), and late-stationary phase (i.e., after 24 h of growth, with OD > 3, hereafter referred as LS). WGBS was also performed on WT cells harvested during SP (OD = 1.5). Moreover, the SMRT methylome of a previously-identified sub-population that is resistant to a cationic AMPs (i.e., polymyxin B^44^) was also investigated. The 8 identified motifs were found in all the methylomes analyzed. For a given motif, a high (>94%) and similar proportion (coefficient of variation <0.01) of methylated sites was observed (Tables 2 and 3). For a given motif identified by SMRT sequencing, the IPD (interpulse duration) ratio did not drastically change over the course of growth (Table 2). Taken together, these results indicate that in all the tested conditions, a high rate of methylation was reached for each of the MTases responsible for the methylation of the 8 identified motifs. This suggests that the corresponding MTase-encoding genes were expressed in all these growth conditions.

### Expression of predicted MTases

**Figure 2:**
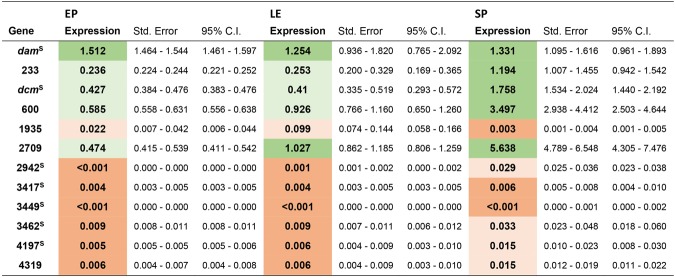
Relative expression levels of MTase genes in *P*. *luminescens* TT01. The fold difference in expression levels of each MTase gene relative to the gene coding for *gyrB* (plu0004) is indicated. Measurements were performed on RNA extracted from 3 independent experiments. EP, exponential phase; LE, late exponential phase; SP, stationary phase. Various colors represent various ranges of level of expression. Green, >1-fold the *gyrB* gene; light green, >0.1-fold the *gyrB* gene. Orange, <0.1-fold the *gyrB* gene; dark orange, <0.01-fold the *gyrB* gene. ^S^Solitary MTase.

The expression level of the 12 MTase-encoding genes was analyzed by qRT-PCR on mRNA extracted from cells grown in LB and harvested during exponential phase (EP and LE) and stationary phase (SP) relative to *gyrB*, a housekeeping gene. Figure 2 shows that the 12 MTases could be split into two groups: MTases (n = 7) encoded by a gene with a low or very low level of expression compared to *gyrB* (less than 10-fold, or 100-fold, respectively) and MTase genes (n = 5) that were expressed at a higher level (>0.1-fold the *gyrB* level of expression). These two groups were identical for the 3 growth conditions tested, revealing that a given MTase gene was expressed at similar levels in the various tested conditions. This is in agreement with the finding that the methylome of TT01 was very similar for all the conditions tested.

Most (5/7) solitary MTase-encoding genes were affiliated to the group with a low level of expression (compared to *gyrB*). Only *dam* and *dcm*, the two broadly conserved solitary MTase-encoding genes, could be assigned to the group with the higher expression (Fig. 2). In contrast, all MTases associated with a RM system except *hsdM* (plu4319), were affiliated to the group with a high level of expression (compared to *gyrB*).

Comparative analysis revealed that none of the 12 MTase-encoding genes was significantly differentially expressed between the LE and EP (Fig. S2A). In contrast, during SP compared to EP, the expression of two and five MTases was significantly downregulated or upregulated, respectively. Among the MTase genes affiliated to the group with a high level of expression, only plu0233 and plu0600 were significantly more expressed during SP compared to EP (Fig. S2B). However, methylome analysis of cells harvested during these two different growth conditions revealed no difference in motif detection, suggesting that a limited increase or decrease in the level of expression of these MTases did not significantly contribute to the genome methylation pattern in *P*. *luminescens* TT01.

Finally, given their very low expression levels (<0.001-fold the *gyrB* gene), five solitary MTases (Plu2942, Plu3417, Plu3449, Plu3462 and Plu4197) could be assumed to be inactive and therefore may not significantly contribute to the genome methylation pattern in *P*. *luminescens* TT01.

### Motif-MTase assignment

Based on *in silico* analysis, 7 of the 8 identified motifs could be assigned to 6 of the MTases found in the genome (Table 1), as follows. The m6A modifications observed in motifs I, III and V could be targeted by the MTases Dam (plu0087, solitary MTase), HsdM (plu4319, RM system) and plu2709 (RM system), respectively; the m4C modifications observed in motif IV could be targeted by plu1935 (RM system); the m5C modifications observed in motifs VI and VII could be targeted by Dcm (plu0338, solitary MTase) and PluTI (plu0599, RM system), respectively (Table 1). Thus, only the m6A modifications observed in motif II could not be associated to an MTase based on *in silico* analysis. Considering the affiliation of plu0233 to the group with the high level of expression (Fig. 2), motif II could be targeted by plu0233 (a hybrid REase-MTase).

### Genome-Wide Analysis of Modification Profiles

All of the 52041 methylated nucleotides found in these 8 methylated motifs were distributed across the genome (Fig. 1). A dedicated webpage with a genome browser displaying the precise position of the methylated nucleotides has been generated (access details can be found in the “Data Availability” section below).

The distribution over the TT01 chromosome of the most prevalent methylation motif (GATC) was analyzed using the DistAMO tool and revealed a heterogenic GATC motif distribution (Fig. S3). As regions with a high GATC density are also regions displaying a high DNA methylation rate, their genomic localization was identified, and their distribution in the core and accessory genome of *P*. *luminescens* was determined. The 38 high-GATC-density regions identified are listed in Table S1. Eighteen were distributed in the core genome, and 20 in the accessory genome as follows: genomic islands (GI, n = 14), regions of genomic plasticity (RGP, n = 4), or phagic regions (n = 2). The major functions associated with these regions were “metabolism” (n = 22), “virulence” (n = 5) and “antibiotic synthesis” (n = 5)^46^.

### Methylation pattern of a clonal subpopulation

We previously identified a small fraction of the *P*. *luminescens* TT01 population that is resistant to cationic AMPs and revealed that this subpopulation is the one responsible for successful infection in insects. Using SMRT sequencing, we showed that this subpopulation was genetically identical to a bacterial population composed of >99% of cells with an AMP-sensitive phenotype^44^. Here, we analyzed the SMRT data on the methylation pattern of the AMP-resistant subpopulation in order to identify the differences in m4C or m6A modification marks compared to the control sample (EP) corresponding to cells with an AMP-sensitive phenotype. The number of modification marks identified was 41114 in the AMP-resistant subpopulation (Table 2) and 41143 in the control sample (EP). For each of the motifs analyzed, a similar proportion (ranging from 96.2% to 100% depending on motif) of methylated sites was observed (coefficient of variation <0.01) between the AMP-resistant subpopulation and the control sample (Table 2). Altogether, only 37 modification marks differed between the two samples, as follows: 26 in motif I, 3 in motif II, 4 in motif III, and 4 in motif IV (detailed data can be found in the genome browser). None of these differential modification marks was located in the vicinity of the genomic regions harboring the *pbgP* operon (encoding enzymes involved in LPS modification and required for the AMP resistance) or the *phoP/phoQ* genes (encoding a two-component system required for the activation of *pbgP* expression). Thus, the global methylome of the AMP-resistant subpopulation analyzed after SMRT sequencing was highly similar to that of the WT grown during the control condition.

### Analysis of the location of unmethylated motifs

**Figure 3:**
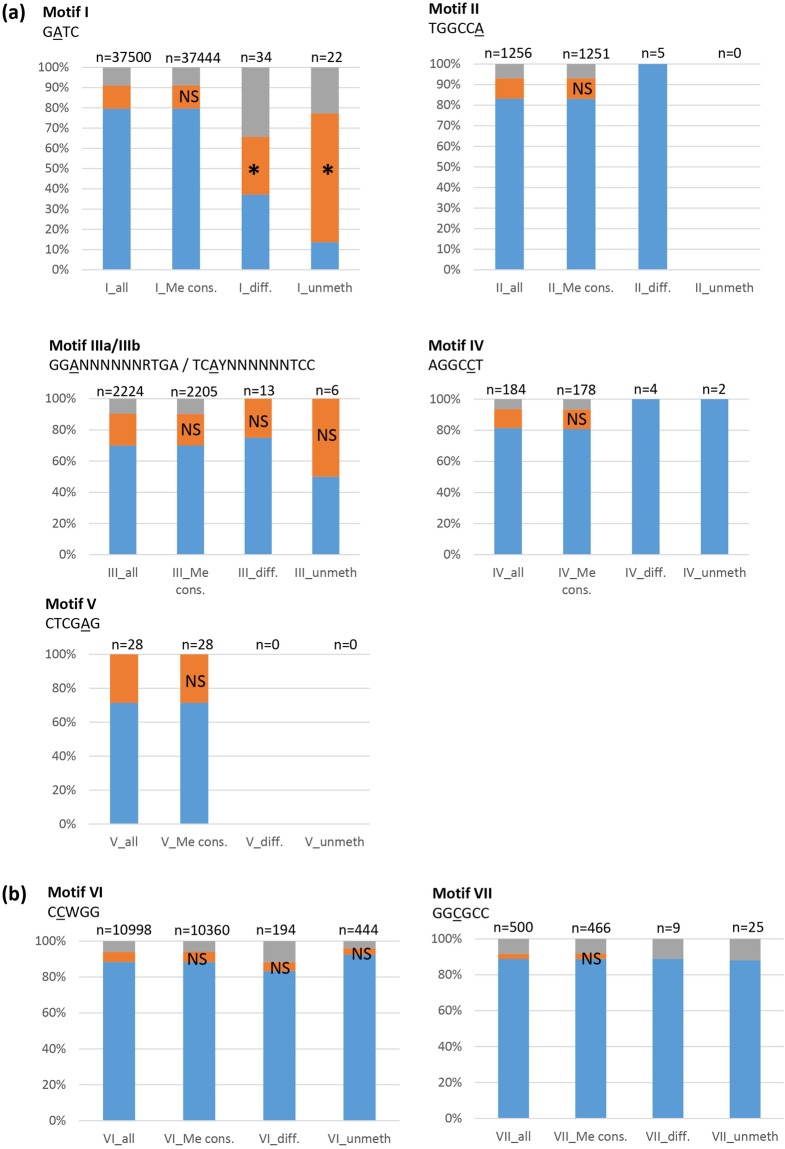
Location of identified motifs in putative gene-regulatory regions. Proportion of motifs identified by SMRT sequencing (**a**) or by WGBS (**b**) located in gene body (blue), in putative promoter regions (i.e. <200 bp upstream from a start codon) (orange), or in intergenic regions (grey). (all), all motifs found in the genome; (cons.), methylated motifs conserved in all of the 4 growth conditions tested (SMRT) or in the 2 growth conditions tested (WGBS); (diff.), methylated motifs in one, two or three of the 4 tested growth conditions (SMRT) or in one growth condition (WGBS); (unmet.), unmethylated motifs conserved in all of the 4 (SMRT) or 2 (WGBS) growth conditions tested. Asterisks indicate that the proportion of motifs located in the upstream region *vs* gene body is significantly different from the proportion observed in the ≪all≫ condition (p < 0.001, Fisher’s exact test). NS, not significant (p > 0.05).

For each of the 8 motifs identified, and for the four growth conditions tested, the number of unmethylated motifs are rare in *P*. *luminescens* (Fig. 3), in agreement with the high fraction of modifications marks identified (Tables 2 and 3). The location of the motif-associated methylation marks was determined relative to the position of neighboring ORFs: either in a putative promoter region (i.e. <200 bp upstream from a start codon), intragenic (inside an ORF), or in other intergenic regions (i.e. >200 bp from a start codon, or downstream of an ORF). For each motif, the fraction of motifs with modification marks (detected in at least one growth condition) mapping to a putative promoter region, as well as the fraction of motif without modification marks (detected in the four growth conditions tested) was calculated. These fractions were compared to the fraction of the corresponding motif mapping to putative promoter regions found in the genome (Fig. 3). For motif II to motif VII, the fraction of unmethylated motifs located in putative promoter regions was not significantly different compared to that observed elsewhere in the genome (i.e. inside an ORF or >200 bp from a start codon, or downstream of an ORF). In contrast, the fraction of unmethylated motif I (in at least one of the four growth conditions tested) located in putative promoter regions was significantly higher than that observed elsewhere in the genome (p < 0.001, Fisher’s exact test) (Fig. 3).

**Figure 4:**
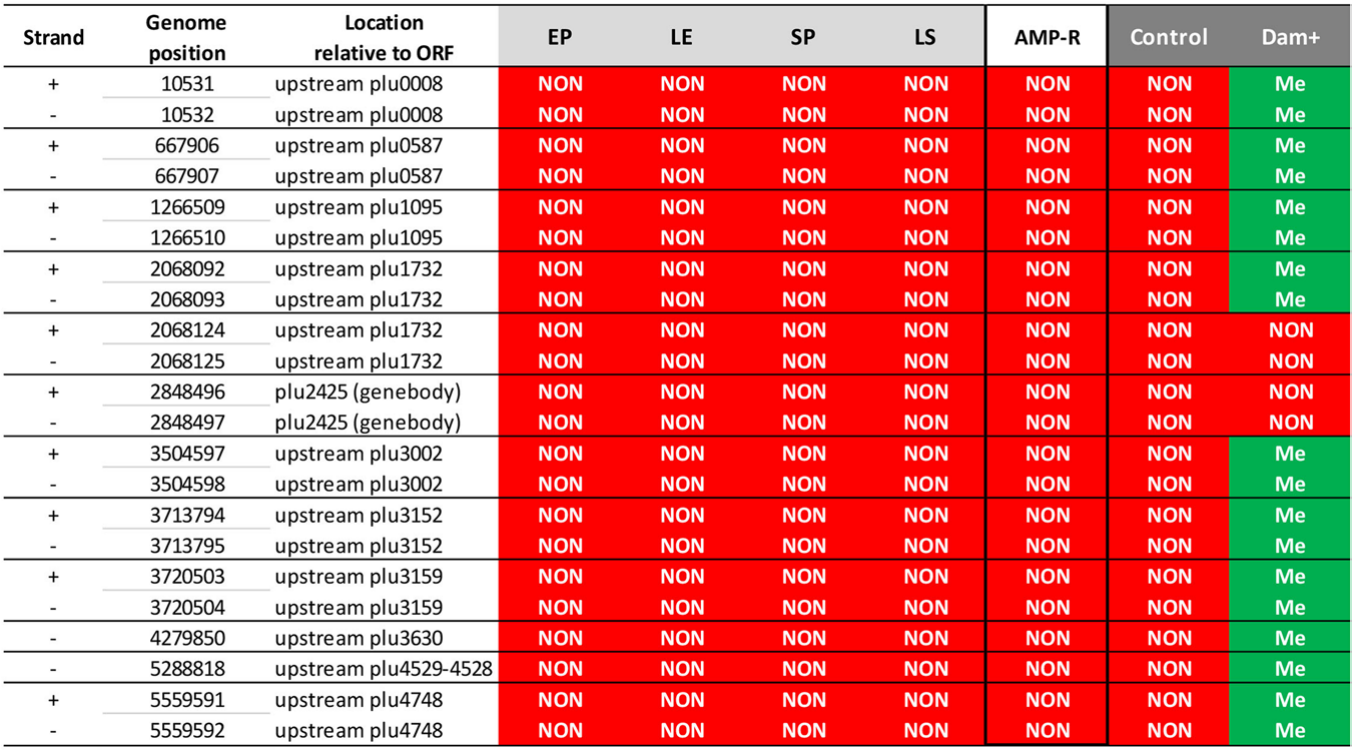
*P*. *luminescens* unmethylated GATC motifs. List of the 22 adenines located in GATC sites which were always unmethylated during the 4 growth conditions tested (light grey). The methylation state of these loci is also indicated for the *P*. *luminescens* AMP-R (i.e. polymyxinB-resistant) subpopulation (white) and for the *P*. *luminescens* strains harboring a plasmid (dark grey). Twenty unmethylated adenines were located upstream from an ORF, and two in a gene body. Red (NON), no modification mark was detected on the adenine of the GATC motif; Green (Me), a modification mark was detected. Note that Dam overexpression (Dam+) restores the DNA methylation of most (18/22) of the adenines located in GATC.

We therefore focused on the precise location of the conserved unmethylated motifs I (GATC). Unmethylated GATC motifs are rare in *P*. *luminescens*: only 56 sites out of 37500 were unmethylated in at least one of the 4 growth conditions tested (Fig. 3). Only a limited number (n = 16) of these unmethylated GATC motifs were located inside an ORF while the majority of them (n = 41) were located either in putative promoter regions, or in other intergenic regions (Fig. 3). As described above, the fraction of these unmethylated GATC motifs located in putative promoter regions was significantly higher than that observed elsewhere in the genome. Among the 56 GATC motifs unmethylated in at least one growth condition, 35 GATC motifs were unmethylated in three or four of the growth conditions tested (detailed data are available in the genome browser). Strikingly, 22 GATC motifs were unmethylated in all four growth conditions tested. Only two were located in a gene body, while the remaining 20 were located in putative promoter regions, with 4 of them mapping to the same promoter region (i.e. upstream plu1732) (Fig. 4). These 22 motifs were distributed all over the chromosome (Fig. 4). Interestingly, all these 22 motifs were still unmethylated in the AMP-resistant subpopulation described above (Fig. 4). Taken together, these results suggest the existence of factors (e.g., DNA binding proteins) hindering DNA methylation at these particular sites. Such factors are presumably always present in the various growth conditions tested, including in the AMP-R subpopulation. No conserved motif, recognizable by transcription factors was observed in these 22 loci based on MEME analysis (data not shown).

### Methylome modifications by Dam-MTase overexpression

We then investigated whether the high proportion (99.8%, Table 2) of the methylated GATC motifs identified in a reference condition could be increased by Dam MTase overexpression. The methylome of a strain overexpressing the Dam MTase was therefore analyzed and compared to its control strain (harboring an empty plasmid).

The number of modification marks identified in the strain overexpressing the Dam MTase and in the control sample (harboring an empty plasmid) was 41175 and 41119, respectively (Table 2). The 68 modification marks differing between the two samples were distributed as follows: 57 in motif I (GATC) which is recognized by Dam MTase, 2 in motif II, 2 in motif IIIa, 4 in motif IIIb and 3 in motif IV (detailed data can be found in the genome browser). As described above, 22 GATC motifs were unmethylated in all four growth-curve time-points tested and were also unmethylated in the control strain harboring an empty plasmid (Fig. 4). In contrast, most of these motifs (n = 18) were found methylated in the strain overexpressing the Dam MTase (Fig. 4). Thus, with only 4 out of 37500 GATC sites found unmethylated in the strain overexpressing the Dam MTase, the proportion of methylated motif I was therefore higher than in the control (99.99% vs 99.83%, respectively). For the other motifs analyzed, a similar proportion of methylated motifs was observed in the strain overexpressing the Dam MTase (coefficient of variation <0.01). Thus, besides an increase in GATC methylation, the global methylome of the strain overexpressing the Dam MTase displayed only a limited number of differences compared to the methylome of the control. These results reveal that Dam overexpression caused a modification of the DNA methylation pattern, which was focused on the unmethylated GATC sites found in the control condition as well as in other various growth conditions tested.

## Discussion

There has been a recent surge in bacterial methylomic data released^32,33,35,37–39,48^. However, in these studies, SMRT sequencing only allowed the analysis of m6A and m4C modification marks, as the thorough identification of the third known DNA methylation mark (i.e. m5C) requires other investigations such as WGBS. Furthermore, most of these studies were performed in only one growth condition. While the genome of *P*. *luminescens* TT01 was sequenced in 2003^45^, our study has only now revealed its complete epigenome (m6A, m4C and m5C) and found no major difference between the various growth conditions tested. Up to now, observation of a relatively stable methylome had only been described for *E*. *coli*^49,50^.

m4C methylation is restricted to archaea and prokaryotes, but in many bacterial species such as *E*. *coli*, no m4C-MTase was identified^32,36,51,52^. In contrast, *P*. *luminescens* displays a clear motif with m4C modification marks (motif IV), with 184 occurrences in the genome at a high rate of methylation (>96% of the motifs are detected as methylated). Based on our results, this motif is presumably methylated by an MTase for which orthologs are mostly found in the *Photorhabdus* genus (plu1935).

Several bacterial variants displaying genetic differences with their ancestor have been analyzed for their DNA methylation pattern (*Haemophilus*, *Neisseria*, *Helicobacter*, *Campylobacter*)^53–56^. In *P*. *luminescens*, we previously described the existence of an AMP-resistant (AMP-R) subpopulation which displays no difference in genome sequence compared to the control population (for which more than 99% of the cells are AMP-sensitive)^44^. The bacterial DNA methylation pattern in a bacterial population grown in presence of antibacterial agents has only been described in *E*. *coli*^49^. Here, we analyzed the methylome of the *P*. *luminescens* AMP-R subpopulation and found that it was highly similar to that of the control population. We thus provide evidence that the AMP used here (i.e. polymyxin B) does not drastically modify the DNA methylation pattern in *P*. *luminescens*. The precise mechanism allowing the AMP-R subpopulation to arise remains unknown, but it requires activation of the expression of the *pbgPE* locus in a PhoPQ-dependent manner^44,57^. In accordance with the high similarity observed between the methylomes of the AMP-R subpopulation and the wild-type control population, no particular modification mark (focused here on m6A and m4C modification marks) mapping to the loci responsible for the AMP resistance could be identified in the AMP-R subpopulation. In addition, Bisulfite sequencing of the promoter region of *pbgPE* genes revealed no m5C difference between both subpopulations (Mouammine & Brillard, unpublished data), suggesting that DNA methylation is not a mechanism triggering the occurrence of the AMP-R subpopulation.

The most prevalent methylation motifs throughout the *P*. *luminescens* genome are GATC (motif I) methylated by Dam, followed by CCwGG (motif VI), methylated by Dcm. Out of the 12 MTases found in *P*. *luminescens* TT01, these two MTases are the only ones for which orthologs are found in numerous bacterial genera other than *Photorhabdus* or the closely-related genus *Xenorhabdus*. As both MTases are widely distributed in *Enterobacteria*, they have been extensively studied^24^. The *P*. *luminescens* methylome analysis also confirmed the presence of a m5C modification mark in motif VII (GGCGCC), a motif previously described as recognized by an RM system (PluTI)^47^. Five additional motifs were also identified in this study, including two not found in REBASE (motifs IIIa and IIIb)^9^.

Here we also show that the Dam methyltransferase is very efficient in *P*. *luminescens*, causing the DNA methylation of more than 99% of the GATC sites in the genome, similarly as what was described in *E*. *coli* or *Salmonella*^5^. Despite this high level of methylation, we identified several loci that were always unmethylated in the tested conditions, suggesting the existence of factors, such as DNA-binding proteins, that hinder these particular GATC sites, as described elsewhere^58^. Strikingly, a significant enrichment of putative promoter regions was observed for these unmethylated motifs but not for the other 6 motifs identified. Dam has been described to act as a regulator of gene expression in *E*. *coli* and *Salmonella* strains, and is therefore considered as involved in epigenetic mechanisms, but such a role in other bacteria remains to be investigated^5^. Our results suggest that in *P*. *luminescens*, Dam may be responsible for a similar mechanism. Furthermore, we provide evidence that Dam overexpression can allow the methylation of these usually-unmethylated sites, suggesting a competition between as-yet-unidentified DNA-binding proteins and the Dam MTase.

MTase overexpression is reported to be related to strong phenotype modifications in several bacterial species, but their methylome has never been investigated^13,15,17,18,59^. In *E*. *coli*, SMRT sequencing of strains overexpressing 3 MTases, for which no particular phenotype was described, revealed signatures in the kinetic variations of the DNA polymerase that were not detected in the parental strain (i.e. methylation of adenines which were not located in a particular motif), suggesting nonspecific activity of these overexpressed MTases^36^. In *P*. *luminescens*, the Dam overexpression was not associated with particular signatures in the kinetic variations of the DNA polymerase compared to the control strain. In contrast, it was associated with an increase in GATC methylation frequency. Further research is required to find out whether this change in the DNA-methylation pattern may be related to the modification of several phenotypes observed in the Dam-overexpressing strain, including impaired motility, impaired virulence in insects, and increased biofilm-forming ability^18^.

## Conclusion

**Figure 5:**
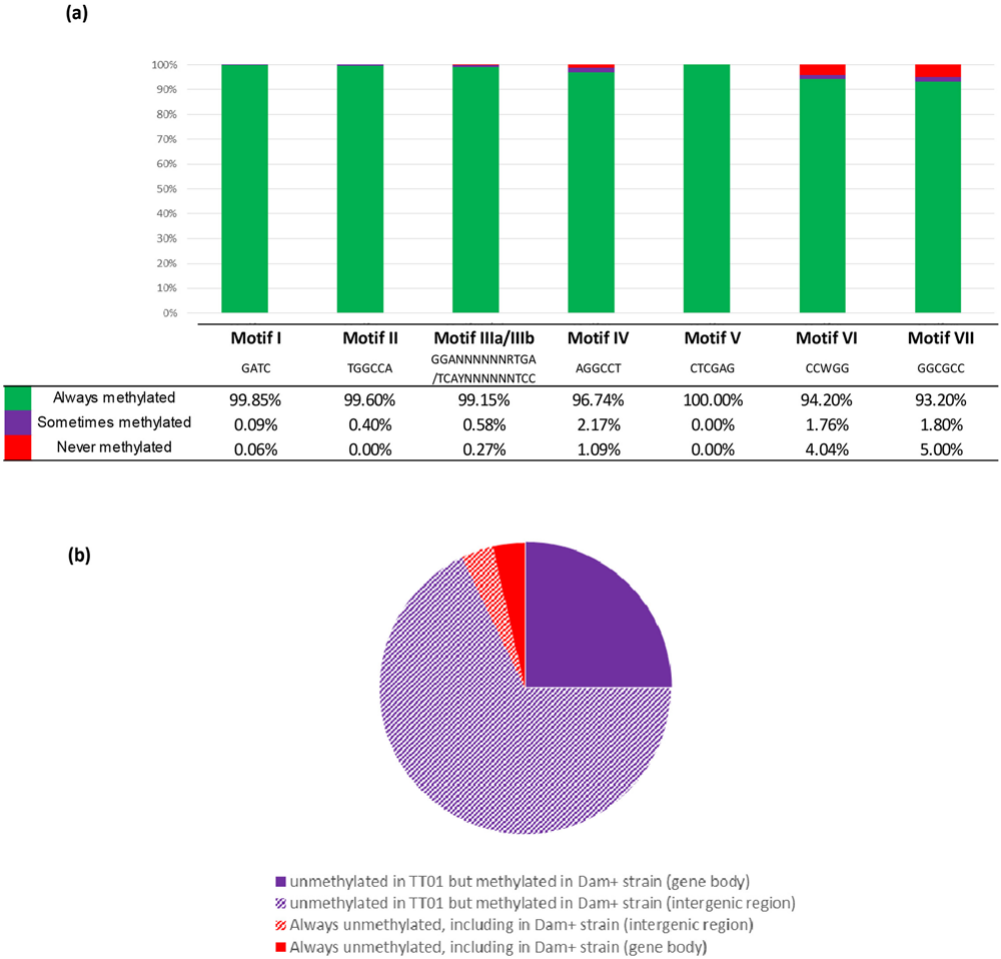
Methylation status of the 8 identified motifs in *P*. *luminescens* TT01. (**a**) For each of the 8 identified motifs, the percentage found methylated in all growth conditions tested (green), in some of the growth conditions tested (purple), or the percentage of motifs found always unmethylated (red) is indicated. (**b**) Location of the 56 GATC motifs which were found unmethylated in at least one of the growth conditions tested in the WT strain (gene body, full color area; intergenic region, hatched area). The methylation status in the *dam*-overexpressing strain is indicated (purple, methylated; red, unmethylated).

This study brings the first description of the methylome of an entomopathogenic bacterium, with the identification of eight motifs displaying a high rate of methylation (Fig. 5). The methylome was stable over growth curve, as well as in an antimicrobial peptide-resistant subpopulation responsible for virulence in insects. The rare unmethylated GATC motifs were located preferentially in putative promoter regions, suggesting that DNA methylation is involved in gene regulation. Overexpression of the Dam MTase can lead to a slight modification of the DNA-methylation pattern, including the methylation of 18/22 sites which are usually protected from methylation by a presumed DNA-binding protein. Given the major modification of phenotypes associated with MTase-overexpression in several bacterial species, the strategy employed here can prove a powerful tool to open a new field of investigation to determine the role of loci protected from DNA methylation in gene regulation.

## Methods

### Strains and growth conditions

The *P*. *luminescens* TT01 bacterial strains were routinely grown in Luria broth (LB) medium with a 180 rpm agitation at 28 °C. As required, antibiotic concentrations used for bacterial selection were gentamycin at 15 μg mL^−1^; polymyxin B (polyB), 100 μg mL^−1^. The AMP-R subpopulation was isolated as previously described^44^. Briefly, addition of polymyxin B to WT cells grown in LB at an OD_540_ of 0.3–0.4 (EP) led to a decrease in OD due to the death of the AMP-sensitive population (about 0.5% of the WT population was found to be AMP-R). The AMP-R cells were then collected when the OD_540_ had returned to 0.3 in the presence of polymyxin B. Genomic DNA was extracted on these cells for methylome analysis.

### Genomic analysis of MTases encoding genes

The REBASE database^9^ was used to identify the putative DNA MTases in the *P*. *luminescens* TT01 genome^45^. The *MaGe* tool (available at https://www.genoscope.cns.fr/agc/microscope/mage/) was used to analyse the genomic context of their encoding genes. The genomic locations were defined in the core or accessory genome (Regions of genomic plasticity (RGP), Genomic Island (GI) and Prophage regions (P)) as previously described (Ogier *et al*.^46^). The *MaGe* tool was also used to analyse gene distribution and their synteny among a panel of *Xenorhabdus* and *Photorhabdus* genomes (27 and 16 strains, respectively, for which a complete genome was available). Distribution of the MTases in other Enterobacterial organisms was performed using a BlastP search on the NCBI non-redundant protein database with default parameters and a maximal target sequences set at 1000.

### Single-molecule real-time (SMRT) DNA sequencing

Genomic DNA was extracted from bacteria grown in LB and harvested at an OD_540_ of 0.3–0.4 (EP), OD = 0.9 (LE), OD = 1.5 (SP), and after 24 h of growth (OD > 3, LS) as follows. Bacterial cells corresponding to 2 ml of culture were washed in PBS and pellets were stored at −80 °C. To perform lysis, cells were resuspended in 200 µl of TSE-lysozyme for 15 minutes at 37 °C, followed by addition of 640 µl EDTA pH8 0.5 M and 160 µl SDS 10% and incubated 15 minutes at 60 °C. Lysates were incubated for 1 hour at 56 °C after addition of 20 µl proteinase K (20 mg·ml^−1^), cooled on ice and incubated 5 minutes at room temperature with 30 µl of RNAse A (20 mg·ml^−1^). Precipitation of contaminants was performed by addition of chilled 350 µl potassium acetate 5 M and a centrifugation step (10,000 × g for 10 min at 4 °C). The genomic DNA was purified with magnetic beads (Sera-Mag Speed beads, Thermo-Scientific) as previously described^60^. The DNA libraries were prepared according to PacBio guidelines: 20-kb Template Preparation Using BluePippin Size-Selection System (15-kb size cutoff); shearing at 40 kb was performed using Megaruptor system (Diagenode); sizing at 17 kb was performed using BluePippin system (Sage). Libraries were sequenced on one or two PacBio SMRT cells at 0.25 nM with the Protocol OneCellPerWell (OCPW), P6C4 chemistry and 360 minutes movies on a Pacific Biosciences RSII instrument (GeT-PlaGe, Toulouse, France). The raw data were processed with the PacBio SMRT Analysis Suite (version v2.3.0 p4). For samples EP, SP and AMP-R, the reads were assembled *de novo*, with the high-quality Hierarchical Genome Assembly Process HGAP.3 algorithm and no rearrangement was observed with progressiveMauve 2.1.0.a1^61^. Conserved Synteny LinePlot revealed 100% conservation of synteny groups between the TT01 genomes studied, with a synton size ≥3 and the *P*. *luminescens* TT01 NC_005126 genome as the reference (data not shown).

### DNA methylation detection and motifs identification after SMRT sequencing

DNA methylation was determined using the RS_Modification_and_Motif_Analysis protocol within SMRT Portal 2.3.0p4 which uses an *in silico* kinetic reference and a Welch’s t-test based kinetic score detection of modified base positions^62^ with parameters set as follow: subread/polymerase read length > = 500, polymerase read quality > = 80 and modification QV > = 30. A score of 30 for the “Modification QV” is the default threshold for calling a position as modified, and corresponds to a p-value of 0.001. Homemade script was used to keep methylated bases for adenine or cytosine with score > = 30 and known motifs.

### Whole Genome Bisulfite DNA sequencing (WBGS) and DNA methylation detection

Genomic DNA from bacteria grown in EP and SP was extracted as described above and was sequenced using Illumina MiSeq technology as previously described^63^.

Fastq files were trimmed for adapters and low quality bases with Trim Galore! (v0.4.4)^64^ then mapped to the public reference genome (NC005126) with Bismark (v0.17.0)^65^. Picard tools were used to remove duplicated reads. Then methylation calling was performed with Bismark_methylation_extractor (v0.17.0) for every single cytosine^65^. Positions at which a sequencing coverage reached 25X or more, and where the proportion of instances that was detected as modified (i.e. number of reads detecting a modification/total number of reads at a given position) reached at least 90% were considered as a methylated base. The surrounding sequences (+/−20 nt) of each methylated cytosine were extracted and analyzed for a motif search using MEME-ChIP (v4.12)^66^. For the 2 motifs identified by WGBS, the mean proportion of reads that was detected as modified at a given position was high (97.3 for motif VI and 96.5 for motif VII). The fraction of motifs methylated was calculated as the number of motifs with a methylated based out of the total number of motifs found in the genome.

### Determination of GATC-rich and GATC-poor regions

DistAMO analysis, which calculate a z-score (with value of 2/-2 considered as a significant value)^67^ revealed heterogenic GATC motif distribution over the TT01 chromosome (Supp. Data Fig. S3). However, the large window sizes used for the calculation of the z-scores of the GATC motif distribution range from 500 kb (at the inner ring) to 50 kb (on the outer ring increasing in 50 kb steps, Fig. S3). In order to have a clearer view of the GATC motif distribution over the TT01 chromosome, we used overlapping windows of a size of 1 kb, sliding every 100 bp. The mean proportion of GATC occurrence found per kb of the whole genome was calculated, and regions of 1 kb displaying at least +/−2 SEM were considered as GATC-enriched or GATC-depleted regions. Such regions were then compared to the position of regions of genomic plasticity previously described^46^.

### RT-qPCR analysis

Total RNA extraction was performed on cells harvested during exponential phase (EP and LE) and stationary phase (SP), from three independent cultures for each strain, using RNeasy miniprep Kit (Qiagen), according to the manufacturer’s instructions. An additional incubation step with DNase I (Qiagen) was performed. The quantity and quality of RNA were assessed with an Agilent 2100 Bioanalyzer with the RNA 6000 Nano LabChip kit. Lack of DNA contamination was controlled by carrying out a PCR on each RNA preparation.

Quantitative reverse transcription-PCR (RT-qPCR) were carried out as previously described^18^. Briefly, RNA samples from 3 biological replicates for each strain were used for cDNA synthesis. The SuperScript II reverse transcriptase (Invitrogen) was used on 1 µg of total RNA with random hexamers (100 ng·µl^−1^; Roche Diagnostics). qPCR analyses were performed using SYBR green Master kit (Roche Diagnostics) with 1 µl of cDNA and specific gene primers at 1 µM (Table S2). The reactions were performed in duplicate at 95 °C for 10 min, followed by 45 cycles at 95 °C for 5 s, 61 °C for 10 s, and 72 °C for 15 s and monitored in the LightCycler 480 system (Roche). Melting curves were analyzed and always contained a single peak. The data analyzed with the REST software 2009^68^ using the pairwise fixed randomization test with 2,000 permutations are presented as a ratio with respect to the reference housekeeping gene *gyrB*, as previously described^18^.

## Electronic supplementary material

Supplementary Information

## Data Availability

The datasets generated and analysed during the current study are available at https://lipm-browsers.toulouse.inra.fr/jbrowse/current/?data=data/private/COLLABORATIONS/photorhabdus-APayelleville-2018/data. SMRT data have also been deposited in REBASE.
