# Supplementary material for: The complete methylome of an entomopathogenic bacterium reveals the existence of loci with unmethylated Adenines
Source: Sci Rep. 2018 Aug 14;8:12091. doi: 10.1038/s41598-018-30620-5 (PMC6092372; doi:10.1038/s41598-018-30620-5)
Supplement: Supplementary file 1 — Supplementary Information [file 41598_2018_30620_MOESM1_ESM.zip › SupplementalData_FigS1.S2.S3.TableS1.S2_2018-05-29.pdf]

The complete methylome of an entomopathogenic bacterium reveals the existence of loci with unmethylated Adenines

Amaury Payelleville[1,4], Ludovic Legrand[2,4], Jean-Claude Ogier[1], Céline Roques[3], Alain Roulet[3], Olivier Bouchez[3], Annabelle Mouammine[1,5], Alain Givaudan[1], and Julien Brillard[1]

[1] DGIMI, INRA, Univ. Montpellier, Montpellier, France

[2] LIPM, Université de Toulouse, INRA, CNRS, Castanet-Tolosan, France.

[3] GeT-PlaGe, INRA, US 1426, Genotoul, Castanet-Tolosan, France

[4] contributed equally to this work

[5] Present address: Department of Fundamental Microbiology, Faculty of Biology and Medicine, University of Lausanne, Quartier UNIL/Sorge, Lausanne, CH1015, Switzerland.

Supplementary data

Supplemental Fig. S1. Genomic context and taxonomic distribution of the 12 methyltransferase (MTases) encoding genes in *P. luminescens* TT01 genome.

Supplemental Fig. S2. Relative expression of the 12 TT01 MTases encoding genes.

Supplemental Fig. S3. GATC distribution over the *P. luminescens* TT01 genome.

Supplemental Table S1: distribution of the high GATC-methyled density regions over the *P. luminescens* TT01 chromosome

Supplemental Table S2. Primers used in this study.

Fig. S1

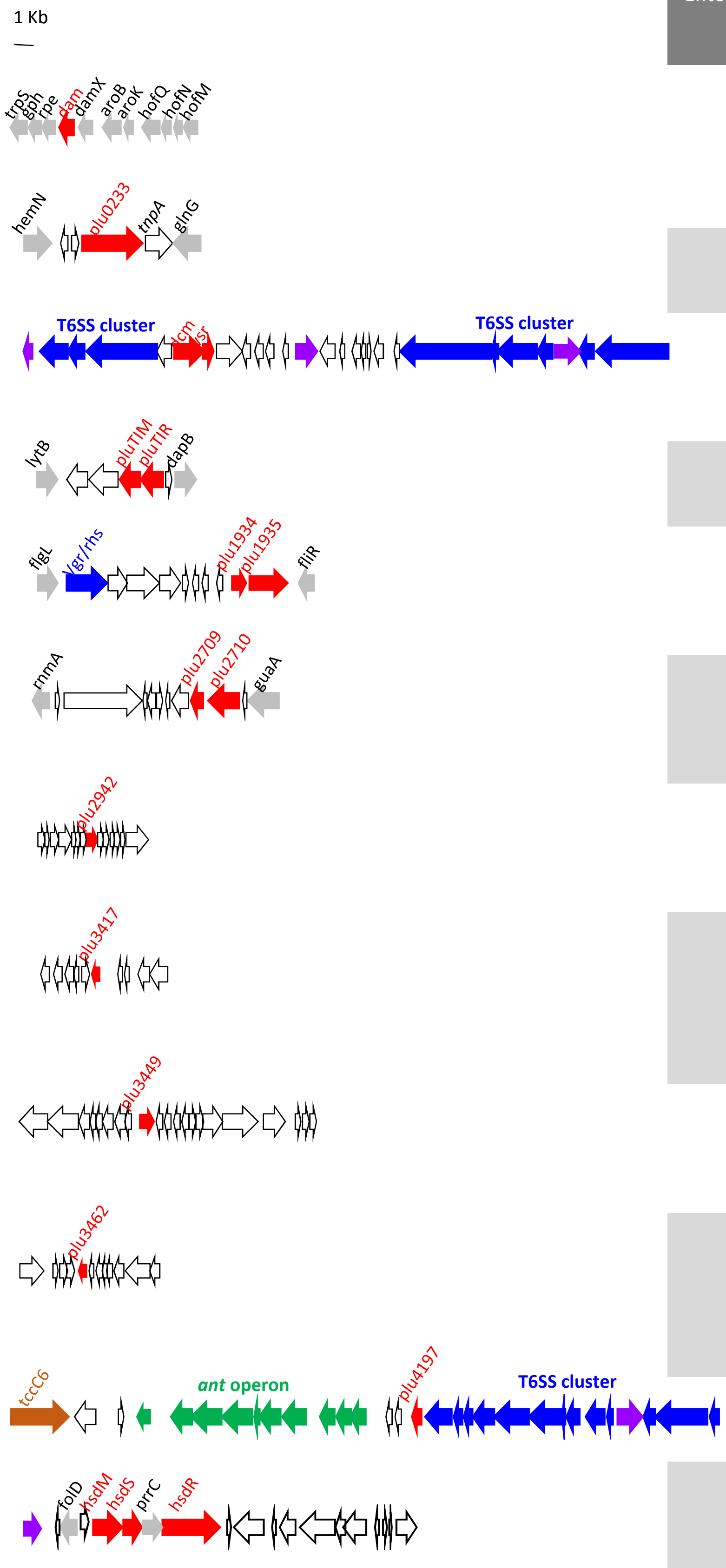

MTase gene	Taxonomic distribution among *Enterobacteriaceae**	Taxonomic distribution among *Photorhabdus* and *Xenorhabdus***
dam ^{S}	++	*Photorhabdus*^{27}(+)<br>*Xenorhabdus*^{16} (+)
plu0233	+/-	*P. luminescens*^{2} (+)<br>*P. temperata*^{2} (+)
dcm ^{S}	++	*Photorhabdus*^{5}<br>*Xenorhabdus*^{7}
plu0599-0600	+/-	*P. luminescens*^{1} (+)<br>*X. bovienii*^{1}
plu1934-1935	w	*P. luminescens*^{2} (+)<br>*P. heterorhabditis*^{1}(+)<br>*P. asymbiotica*^{1} (+)
plu2709-2710	+/-	*P. luminescens*^{1} (+)<br>*P. luminescens*^{1} (+)
plu2942 ^{S}	+	*X. bovienii*^{7} ; *X. species*^{1};<br>*X. poinarii*^{1}
plu3417 ^{S}	+	*P. luminescens*^{5}<br>*P. temperata*^{2}<br>*P. heterorhabditis*^{1}<br>*Xenorhabdus*^{19}
plu3449 ^{S}	++	*P. luminescens*^{4} (+)<br>*P. temperata*^{2} (+)<br>*Xenorhabdus*^{2} (+)
plu3462 ^{S}	+	*P. luminescens*^{5}<br>*P. temperata*^{2}<br>*P. heterorhabditis*^{1}<br>*Xenorhabdus*^{2}
plu4197 ^{S}	w	*P. luminescens*^{1} (+)
hsd	+	*P. luminescens* ^{4} (+)<br>*P. temperata*^{3} (+)<br>*P. asymbiotica*^{2} (+)<br>*X. griffinae*^{1}; *X. species*^{2}

Fig. S1. Genomic context and taxonomic distribution of the 12 methyltransferase (MTases) encoding genes in *P. luminescens* TT01 genome. The gene names (labels) of the MTases are on the left of the figure. ^{S} solitary MTases. Arrows represent individual ORFs, and the names of the genes (or cluster of genes) are indicated above. Red arrows represent genes encoding MTases proteins or their cognate partners, white arrows represent genes encoding proteins of unknown function, purple arrows represent genes encoding transposases, brown arrow represent a gene encoding Insecticidal toxin complex protein, blue arrows represent genes encoding component proteins of type 6 Secretion Systems, green arrows represent genes encoding proteins involved in anthraquinone biosynthesis (type II polyketide synthase) and grey arrows represent housekeeping genes. *Tax BlastP report: the number of Enterobacterial organisms displaying significant hits with the input query is classified as ++>100; 50>+>100; 10>+/->50; w<10. ** The number of *Photorhabdus* and *Xenorhabdus* genomes with orthologous genes is indicated; (+) indicates that orthologous genes are syntenic

Fig. S2

(A) Differential gene expression between EP (OD 0.3) and LE (OD 0.9) relative to *gyrB*

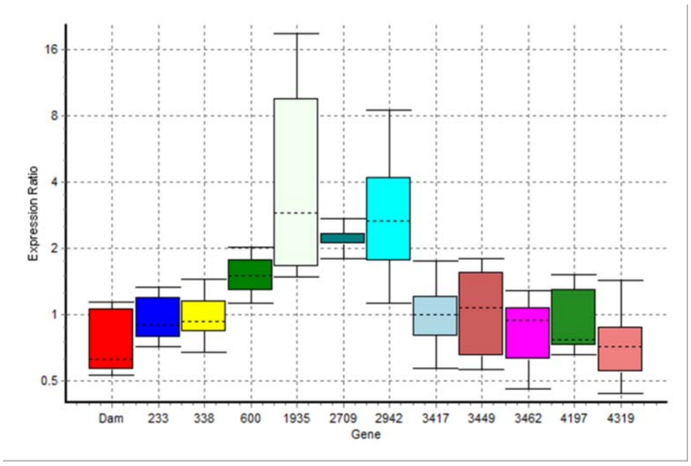

Gene	Expression	Std. Error	95% C.I.	P(H1)	Result
dam	0.72	0.558 - 1.121	0.536 - 1.137	0.299	NS
plu0233	0.95	0.756 - 1.253	0.719 - 1.323	0.606	NS
dcm	0.97	0.777 - 1.259	0.693 - 1.413	0.813	NS
plu0600	1.51	1.272 - 1.956	1.158 - 2.032	0.088	NS
plu1935	3.66	1.588 - 10.037	1.498 - 17.172	0.063	NS
plu2709	2.25	2.034 - 2.608	1.838 - 2.731	0.092	NS
plu2942	2.95	1.732 - 5.897	1.250 - 8.085	0.081	NS
plu3417	1.00	0.733 - 1.372	0.596 - 1.692	0.846	NS
plu3449	1.03	0.582 - 1.575	0.561 - 1.753	0.732	NS
plu3462	0.85	0.596 - 1.175	0.485 - 1.269	0.648	NS
plu4197	0.92	0.706 - 1.421	0.666 - 1.508	0.738	NS
hsdM	0.74	0.518 - 0.985	0.453 - 1.356	0.207	NS

(B) Differential gene expression between EP (OD 0.3) and SP (OD 1.5) relative to *gyrB*

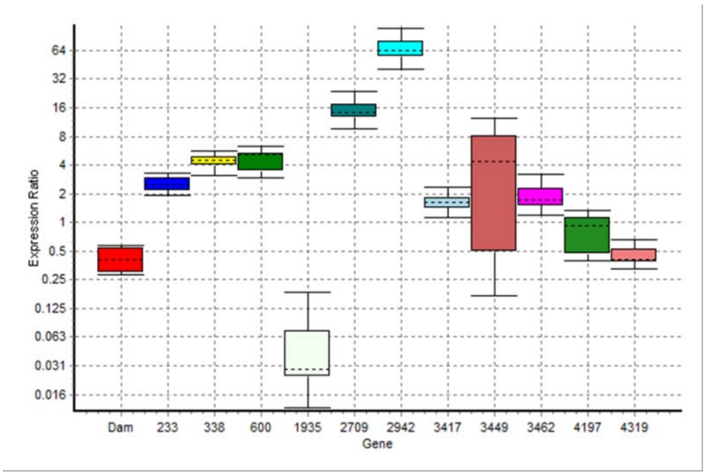

Gene	Expression	Std. Error	95% C.I.	P(H1)	Result
dam	0.41	0.301 - 0.571	0.289 - 0.580	<0.001	DOWN
plu0233	2.53	2.068 - 3.111	1.965 - 3.285	<0.001	UP
dcm	4.31	3.591 - 5.066	3.199 - 5.508	0.072	NS
plu0600	4.60	3.359 - 6.037	3.058 - 6.271	0.036	UP
plu1935	0.04	0.016 - 0.131	0.012 - 0.181	0.058	NS
plu2709	14.89	11.314 - 19.834	9.868 - 23.018	0.072	NS
plu2942	65.20	45.050 - 82.889	40.628 - 103.054	<0.001	UP
plu3417	1.63	1.343 - 1.994	1.166 - 2.272	<0.001	UP
plu3449	2.58	0.368 - 10.893	0.202 - 12.320	0.295	NS
plu3462	1.85	1.381 - 2.800	1.213 - 3.129	<0.001	UP
plu4197	0.80	0.449 - 1.265	0.410 - 1.343	0.658	NS
hsdM	0.44	0.349 - 0.547	0.330 - 0.645	<0.001	DOWN

Fig. S2. Relative expression of the 12 TT01 MTases encoding genes.
qRT-PCR was performed and analyzed as previously described (Mouammine et al., 2017) .
The data for each sample are expressed relative to the level of the control gene (*gyrB*) , using REST software 200968. This method quantified the expression of a target gene relative to that of a reference gene, for comparisons between growth conditions. Samples were up- or down-regulated when P(H1)<0.05, and were considered not significantly different (NS) when P(H1)>0.05.

Fig. S3

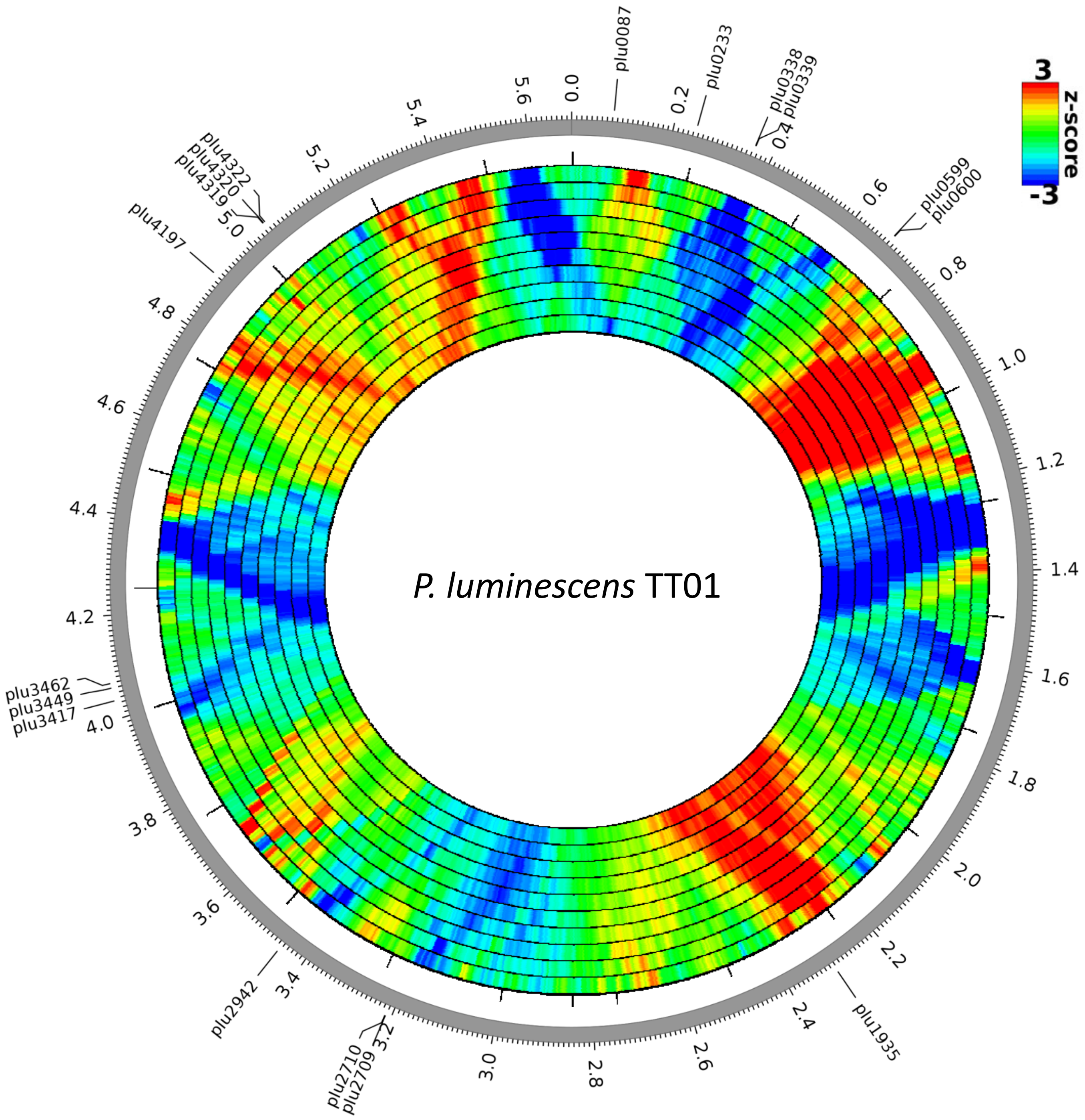

Fig. S3. GATC distribution over the *P. luminescens* TT01 genome.
The GATC distribution was analyzed using DistAMO tool (Sobetzko et al., 2016).
The motif distribution is color-coded as indicated by the legend on the right. A z-score of 2/-2 is commonly accepted as a significant value. The different rings show the distribution using various window sizes for the calculation of the z-scores ranging from 500 kb at the inner ring to 50kb on the outer ring increasing in 50kb steps.

Supplemental Table S1: distribution of the high GATC-methyled density regions over the *P. luminescens* TT01 chromosome

start	end	Localisation	Associated genes	Functional annotation of the associated genes	Functional role of the associated genomic region
42700	45000	Core genome	*gidA*<br>*mioC*	Glucose inhibited division protein A<br>Protein involved in modulation of initiation at oriC	Metabolism
149100	150100	Genomic Island	*plu0142*	putative NADP-dependent aldehyde dehydrogenase	Metabolism
183400	184400	Genomic Island	*yiaO*<br>*yiaM*	Putative permease<br>Putative sugar transporter	Metabolism (transport)
318100	319100	Core genome	*dppA*	Periplasmic dipeptide transport protein	Metabolism (transport)
319200	320200	Core genome	*dppB*	Periplasmic dipeptide transport protein	Metabolism (transport)
394200	395400	Genomic Island	*plu0367*	component of the TSS 6	Microbial Competition / Virulence against insects
505800	506800	Core genome	*malP*	maltodextrin phosphorylase	Metabolism
			malQ	4-alpha-glucanotransferase (amylomaltase)	Metabolism
578600	579700	Core genome	*deoD*<br>*plu0523*	purine-nucleoside phosphorylase (inosine phosphorylase) (PNP)<br>putative cystathionine gamma-lyase	DNA metabolism
636500	637700	Core genome	*thrA*	aspartokinase I-homoserine dehydrogenase I	Metabolism
684500	685500	Core genome	*carA,carB*	carbamoyl-phosphate synthase	Metabolism
692700	693800	Core genome	*pdxA*<br>*surA*	4-hydroxythreonine-4-phosphate dehydrogenase<br>Survival protein SurA precursor (chaperone)	stress resistance
787800	788900	Core genome	*dnaE*	DNA polymerase III alpha subunit	DNA metabolism
964100<br><br>972300	965500<br><br>973800	Genomic Island	*agaR*<br>*kbaZ*<br>*plu0840*<br>*plu0841*	Putative *aga* operon transcriptional repressor<br>D-tagatose-1,6-bisphosphate aldolase subunit KbaZ<br>putative enterotoxin<br>putative amidase	Antibiotic resistance<br>Metabolism
1001600	1002600	Core genome	*pcnB,yadB*	Poly(A) polymerase I<br>Glutamyl-Q tRNA(Asp) synthetase	Metabolism
1009900	1010900	Core genome	*mrcB*	penicillin-binding protein 1B	Antibiotic resistance
1091700<br><br>1172100	1092700<br><br>1173100	Genomic Island	*plu0949*<br>*plu0950*<br>*hpaE*<br>*hpaG2*	putative transcriptional regulator<br>FAD/FMN-dependent dehydrogenase<br>5-carboxymethyl-2-hydroxymuconate semialdehyde dehydrogenase<br>4-hydroxyphenylacetate degradation bifunctional isomerase/decarboxylase	Metabolism
1702400	1703400	Core genome	*kdpD*	sensor protein (histidine kinase)	Metabolism
1956200	1957200	Core genome	*mukB*<br>*mukE*	Chromosome partition protein MukB	Cell division
2034700	2035700	Phagic region	*plu1703,plu1704,plu1705*	Phagic genes	Phage components
2532200	2533200	Core genome	*azoR*<br>*hrpA*	Azoreductase, NADH-dependent<br>ATP-dependent helicase	Metabolism
3096500	3097500	Region of Genomic Plasticity	*plu2643*<br>*plu2644*	putative MFS transporter<br>putative peptide synthetase	antibiotic synthesis
3129200<br>3151500	3130300<br>3152500	Region of Genomic Plasticity	*plu2670*<br>*plu2670*	putative peptide synthetase<br>putative peptide synthetase	antibiotic synthesis
3212900	3213900	Region of Genomic Plasticity	*plu2710*<br>*plu2711*	DNA methyltransferase<br>Putative recombinase	Recombination genes
3492300	3493300	Phagic region	*cbiH*<br>*cbiJ*	precorrin-3 methyltransferase<br>Synthesis of vitamin B12 adenosyl cobalamide	metabolism (vitamin B12 biosynthesis cluster)
3626700	3627700	Genomic Island	*astB*<br>*astE*	succinylarginine dihydrolase<br>Succinylglutamate desuccinylase	metabolism
3671000	3672300	Genomic Island	*mcf2*	Mcf2 cytotoxin (insecticidal toxin)	virulence against insects
3684800	3685800	Genomic Island	*plu3130*	putative non-ribosomal peptide synthetase	antibiotic synthesis
3860600	3861800	Genomic Island	*plu3260*	putative Type VI secretion protein	Microbial Competition / virulence against insects
4436100	4437600	Genomic Island	*sctO,sctP,sctQ*	Type III secretion component proteins	virulence against insects
4516800	4518200	Core genome	*plu3845*<br>*priC*<br>*ybaM*	iron-sulfur binding protein<br>primosomal replication factor N<br>Protein of unknown function	metabolism
4746300	4747300	Core genome	*tldD*	Metalloprotease	metabolism
4750800	4751800	Core genome	*yhdP*	Protein of unknown function	unknown
4818600	4819600	Core genome	*efp,yjeK*	elongation factor P<br>putative L-lysine 2,3-aminomutase	metabolism
4884600	4885600	Genomic Island	*plu4179*	putative the phenylacetyl-CoA ligase	metabolism (phenylacetic acid catabolic pathway)
4947200	4948300	Genomic Island	*plu4226,plu4227*	Putative Type VI secretion proteins	Microbial Competition / virulence against insects

Table S2. Primers used in this study.

Oligonucleotides	use	Sequence (5'- 3')
F-0004	qRT-PCR on *gyrB* gene	ATACACGAAGAAGAAGGTGTTTCAG
R-0004	qRT-PCR on *gyrB* gene	TACCTGTCTGTTCAGTTTCTCCAAC
F-0087	qRT-PCR on *dam* gene	CTGATGAGTTTATAGTCCACACACG
R-0087	qRT-PCR on *dam* gene	ACCATGAGAATTATAACGACAGAGG
F-0600	qRT-PCR on plu0600 gene	ACTCGTCTGATAGCAGAGATGAAAG
R-0600	qRT-PCR on plu0600 gene	GAGTAATGGCATCTACAGCATAACC
F-0233	qRT-PCR on plu0233 gene	CATGGTGATCAGATTCATACTCAAC
R-0233	qRT-PCR on plu0233 gene	AGTAAAGCAAACCATCAGACATAGC
F-0338	qRT-PCR on plu0338 gene	AACAGAAGTTGAAGTTGCTATGCTC
R-0338	qRT-PCR on plu0338 gene	AATACCAGTTTGCTTTGTAGGTACG
F-1935	qRT-PCR on plu1935 gene	AGCTTGGAGCAAGTATTATAGGATG
R-1935	qRT-PCR on plu1935 gene	ATCGGGAACCATTGTTGTATAGTAG
F-2710	qRT-PCR on plu2710 gene	AGACCTTTAAGAGCACCAAAGCTG
R-2710	qRT-PCR on plu2710 gene	GGTAACGACTACGATATTCTGCAAG
F-2942	qRT-PCR on plu2942 gene	CTTCGATTTAACTAAACTCCCCATC
R-2942	qRT-PCR on plu2942 gene	GTTTTTATCTATCCGATCACCAGTC
F-3417	qRT-PCR on plu3417 gene	CAATTACCCAATGAAGTGGATTACC
R-3417	qRT-PCR on plu3417 gene	CCATATTCCAGACTCAATTCCTCAT
F-3449	qRT-PCR on plu3449 gene	GATCTTAATGGTGAGTTGGTGAATC
R-3449	qRT-PCR on plu3449 gene	GCCTGCTTTTGGAGATAGTAGAAAC
F-3462	qRT-PCR on plu3462 gene	AAAACGTTACCAGACAACTGCATAG
R-3462	qRT-PCR on plu3462 gene	TATATACAAACTGCCGTTGGGTTTC
F-4197	qRT-PCR on plu4197 gene	CTAACGTAGCCCGTTTTATGACATC
R-4197	qRT-PCR on plu4197 gene	GTTATAGAACTCTCGCACCCAGTTC
F-4319	qRT-PCR on plu4319 gene	ATAGATCTGTTTGGTGATGCTTACG
R-4319	qRT-PCR on plu4319 gene	TTTTGGCTTGTAATAGCAGAGAGC
